# Unveiling sialoglycans’ immune mastery in pregnancy and their intersection with tumor biology

**DOI:** 10.3389/fimmu.2024.1479181

**Published:** 2024-12-20

**Authors:** Jianmei Huang, Lu Feng, Jianming Huang, Guonan Zhang, Shixiu Liao

**Affiliations:** ^1^ Medical Genetic Institute of Henan Province, Henan Key Laboratory of Genetic Diseases and Functional Genomics, National Health Commission Key Laboratory of Birth Defects Prevention, Henan Provincial People’s Hospital, People’s Hospital of Zhengzhou University, Zhengzhou University, Zhengzhou, Henan, China; ^2^ Department of Oncology, The Affiliated Hospital of Southwest Medical University, Luzhou, Sichuan, China; ^3^ Biochemistry and Molecular Biology, Sichuan Cancer Institute, Chengdu, China; ^4^ Department of Gynecologic Oncology, Sichuan Cancer Hospital, School of Medicine, University of Electronic Science and Technology of China, Chengdu, China

**Keywords:** sialoglycan, glyco-immune checkpoint, pregnancy, maternal-fetal immunity, tumor, immunity

## Abstract

Sialylation is a typical final step of glycosylation, which is a prevalent post-translational modification of proteins. Sialoglycans, the products of sialylation, are located on the outmost of cells and participate in pivotal biological processes. They have been identified as glyco-immune checkpoints and are currently under rigorous investigation in the field of tumor research. It is noteworthy that the exploration of sialoglycans in tumor and pregnancy contexts was both initiated in the 1960s. Mechanisms in these two conditions exhibit similarities. Trophoblast infiltration during pregnancy gets controlled, while tumor invasion is uncontrolled. The maternal-fetal immunotolerance balances acceptance of the semiallogeneic fetus and resistance against “non-self” antigen attack simultaneously. Tumors mask themselves with sialoglycans as “don’t eat me” signals to escape immune surveillance. The trophoblastic epithelium is covered with sialoglycans, which have been demonstrated to play an immune regulatory role throughout the entire pregnancy. Immune abnormalities are commonly recognized as an important reason for miscarriages. Therapeutic strategies that desialylation and targeting receptors of sialoglycans have been studied in tumors, while agents that target glyco-immune checkpoints have not been studied in pregnancy. Thus, investigating the roles of sialoglycans in pregnancy and their intersection with tumors may facilitate the development of novel therapies targeting glyco-immune checkpoints for the treatment of pregnancy-related diseases, such as miscarriage and preeclampsia.

## 1 Introduction

The outermost layer of all cells is decorated with glycocalyx coatings. Sialic acid usually serves as the terminal monosaccharide of the glycocalyx. This outermost location of sialic acid makes it a critical role in mediating cellular connections between cells and the extracellular matrix. Sialic acid-containing glycans (sialoglycans) are engaged in various cellular biological processes (1) and usually combine with sialic acid-binding immunoglobulin-like lectins (Siglecs). Recent research has elucidated notable roles played by the interactions between sialoglycans and Siglecs in immune responses, thus recognizing them as crucial glyco-immune checkpoints (2, 3).

The investigation of sialoglycans in tumor immunity is now being conducted widely and rapidly. However, it is noteworthy that around the 1960s, sialic acid was concurrently recognized as a significant determinant in both tumor (4) and pregnancy (5). In these contexts, cells must evade immune surveillance—trophoblasts during gestation and tumor cells during metastatic progression—by manipulating immune checkpoint pathways. The underlying mechanisms of these two conditions are somehow similar. Tumor cells and trophoblastic epithelium are both covered with sialoglycans to escape immune attack (5, 6). This review aims to investigate the intersection of immune regulatory roles of sialoglycans in pregnancy and tumors, pointing out their potential as therapeutic targets. In order to enhance comprehension of the functions of sialoglycans in pregnancy, we investigated their roles throughout the entirety of the gestational period, as well as their overlap relevance in tumor immunity.

## 2 The sialoglycan biosynthesis

**Figure 1:**
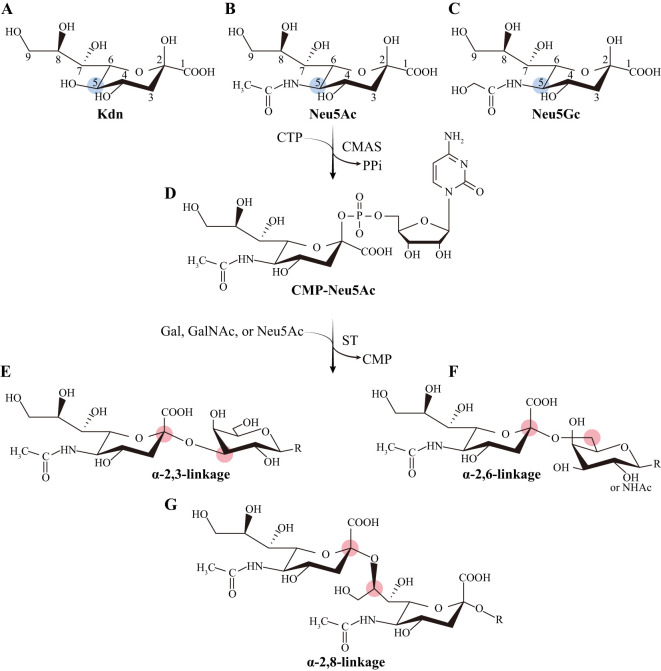
The predominant forms of sialic acids. **(A)** Kdn is the backbone of the family of sialic acids. **(B)** Neu5Ac and **(C)** Neu5Gc are the most abundant forms of sialic acids, and the former is the predominant form in humans. **(D)** CMP-Neu5Ac is the activated form of Neu5Ac. **(E–G)** Three major glycosidic bonds of sialoglycans. Kdn: neuraminic acid or deaminoneuraminic acid; Neu5Ac: N-acetylneuraminic acid; Neu5Gc: N-glycolylneuraminic acid; CMP-Neu5Ac: The cytidine 5’-monophosphate-sialic acid. The relevant chemical structures were reconstructed based on references (1, 7–9).

Sialic acids comprise a collection of more than 50 sugars with nine carbon atoms, which are derived from neuraminic acid or deaminoneuraminic acid (Kdn, **Figure 1A**). The most prevalent types of sialic acids are N-acetylneuraminic acid (Neu5Ac) and N-glycolylneuraminic acid (Neu5Gc), and the former one is the predominant form in humans (**Figures 1B, C**) (7). Neu5Ac is subsequently carried into the nucleus and forms its activated form, cytidine 5’-monophosphate-sialic acid (CMP-Neu5Ac, **Figure 1D**), by the enzyme CMP-Neu5Ac synthase (CMAS) (8, 9). The Neu5Ac moiety of CMP-Neu5Ac is transferred to the termini of glycoproteins and glycolipids by sialyltransferases (9, 10). The process of attaching sialic acids to the terminals of glycocalyx is referred to as sialylation. Enzymes known as β-galactoside α-2,3-sialyltransferases (ST3Gals), β-galactoside α-2,6-sialyltransferases (ST6Gals), and α-N-acetylgalactosaminide α-2,6-sialyltransferases (ST6GalNAcs) catalyze the attachment of sialic acids to galactose (Gal) or N-acetylgalactosamine (GalNAc) through an α-2,3/6-linkage (**Figures 1E, F**). Alpha-2,8-sialyltransferases (ST8Sias) catalyze the synthesis of polysialic acids (**Figure 1G**). Sialoglycans are divided into three classes based on the types of above glycosidic bonds: α2,3-sialoglycans, α2,6-sialoglycans, and α2,8-sialoglycans (**Figures 1E–G**). The enzymatic process of sialylation altered the glycocalyx of cells, affecting cell-cell interactions essential for immune tolerance during pregnancy and tumor immune evasion.

## 3 Sialoglycan-mediated maternal-fetal interaction

**Figure 2:**
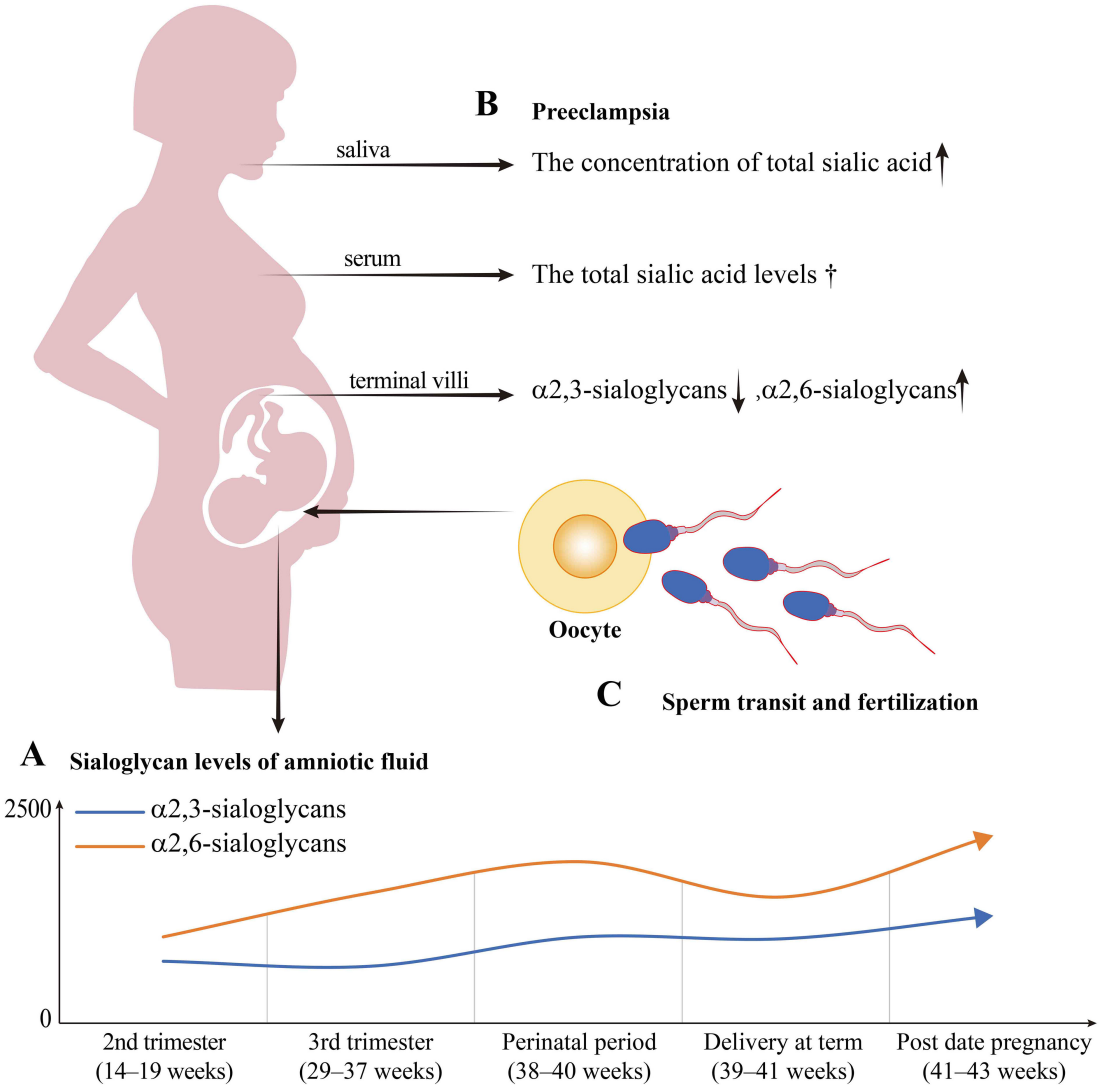
The sialoglycan level trends in normal pregnancy period and preeclampsia. **(A)** The sialoglycan level curves in normal pregnancy amniotic fluid were simulated based on the results of normal pregnant women by Magdalena et al (14). The α2,3-sialoglycans refer to the glycans detected by Maackia amurensis (MAA). The α2,6-sialoglycans refer to the glycans detected by Sambucus nigra (SNA). The relative amounts of α2,3-sialoglycans and α2,6-sialoglycans were positively related to the age of pregnancy. The α2,3-sialoglycans and α2,6-sialoglycans both reach the highest point during the post-date pregnancy. The α2,3-sialoglycans are significantly elevated in post-date pregnancy compared to the 3rd trimester (the blue curve). The α2,6-sialoglycans gradually rise from the 2nd trimester to the perinatal period, and thereafter decrease to a level similar to that of the 3rd trimester during delivery (the orange curve). **(B)** The sialic acid levels change while preeclampsia. Compared to the normal pregnancy, the concentration of total sialic acid in the saliva significantly increased; the total sialic acid levels in serum showed no significant difference (marked with †); levels of α2,6-sialoglycans in the syncytium elevated, while that of α2,3-sialoglycans in the endothelium of terminal villi decreased. **(C)** Sialoglycans play roles in gamete transit and fertilization. The negatively charged sialoglycan coat of sperm renders them to escape the immune-mediated clearance and assists in sperm maturation and fertilization within the FRT.

Maintaining the maternal-fetal immunity balance is crucial for a successful pregnancy (11). Endogenous sialoglycans, which hinder leukocyte activation via “self-associated molecular patterns” (SAMP) (12) are pivotal for the protection of the semi-allogeneic embryos. The deficiency of sialoglycans due to the genetic removal of CMAS lead to the death of embryos at around 9.5 days after fertilization in mice (13). As shown in **Figure 2A**, amniotic fluid concentrations of α2,3-sialoglycans and α2,6-sialoglycans are positively related to gestational weeks, suggesting their critical role in maintaining fetal tolerance (14). When complicated with preeclampsia, the concentration of total sialic acids in the saliva of pregnant women exhibited a considerable rise (15); α2,3-sialoglycans concentrations decreased in the syncytiotrophoblast and fetal endothelium of the placental terminal villi (16); levels of α2,6-sialoglycans in the syncytium elevated (17); but the serum levels of sialic acid were not significant changed (18) (**Figure 2B**). A key pathogenic factor in the development of preeclampsia is the impairment of maternal immune tolerance towards the semiallogeneic fetus (19). It is remains to be revealed if alterations in sialic acid levels in preeclampsia contribute to this immunological imbalance. Moreover, sialoglycans may commence to act even before conception (**Figure 2C**). The follicular fluid of women who successfully achieved pregnancy through *in vitro* fertilization may contain higher levels of total sialic acids than those who were unsuccessful (*p*=0.064; significance was not achieved) (20). Furthermore, sialoglycans are necessary for sperm and embryos to survive in the female reproductive tract (FRT) until delivery (21–24).

### 3.1 Effects on gamete transit and fertilization

**Figure 3:**
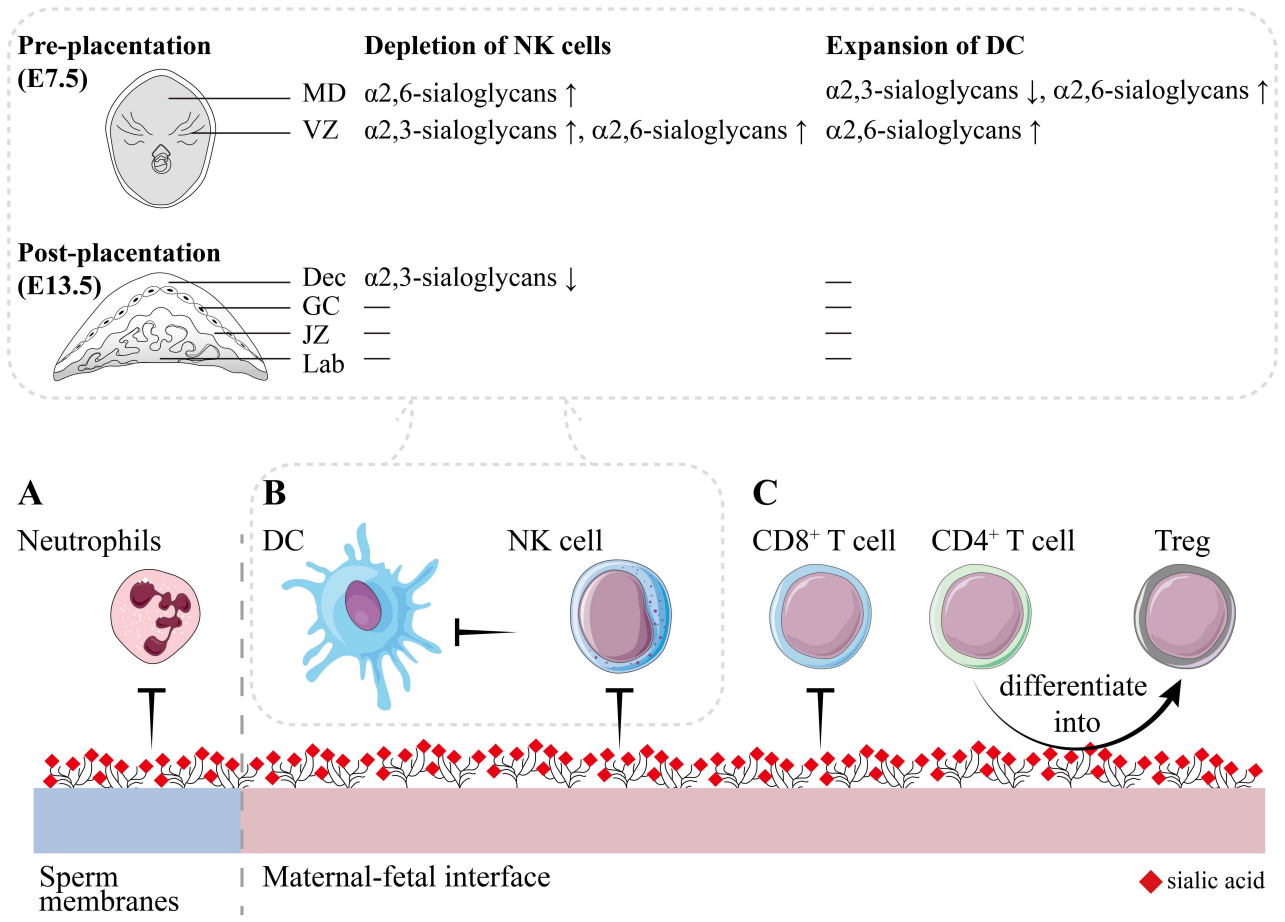
The immune regulatory roles of sialoglycans during pregnancy. **(A)** The immunological regulation of the FRT initiates when sperm and seminal fluid come into touch. The presence of sialoglycans on sperm inhibits the leukocytic response, hence promoting the viability of sperm within the FRT. **(B)** The balanced DC-NK cell interactions are critical during the placentation stage. During pregnancy, sialoglycans protect allogeneic embryos against NK immunosurveillance and NK-mediated killing. Furthermore, this dysfunction of NK cells hinders the activation of DC. While DC undergoes expansion during the early stages of pregnancy, sialoglycans have the capacity to impede its activation and immunogenicity. Compared with normal pregnancy, during the pre-placentation stage (embryonic day 7.5, E7.5), depletion of NK cells increases the α2,6-sialoglycans in MD; increases α2,3-sialoglycans and α2,6-sialoglycans in VZ. Expansion of DC decreases α2,3-sialoglycans and increases α2,6-sialoglycans in MD; increases α2,6-sialoglycans in VZ. During the post-placentation stage (E13.5), depletion of NK cells decreases the α2,3-sialoglycans52. **(C)** T cells remain in a state of suppression. Sialoglycans suppress the cytotoxicity of CD8+ T cells and induces the differentiation of CD4+ T cells into Treg. MD, mesometrial decidua; VZ, vascular zone; Dec, decidua; GC, giant cells; JZ, junctional zone; Lab, labyrinth. Embryo schematic diagram originated from reference (52).

The immune modulation of the FRT commences upon the contact of sperm and seminal fluid to guarantee successful pregnancy (**Figure 3A**) (21). Sperm-associated sialoglycans impede the leukocytic reaction by interacting with Siglecs on the endometrium, hence facilitating the survival of sperm in the FRT (22). The sialoglycans expressed on surviving sperm engage with Siglecs, leading to the induction of immune suppression within the environment of the oviduct (25). Spermatozoa also impacts fertilization by modulating the immunological response through regulating the chemokine, growth factor, and cytokine expression in the fallopian tube epithelial cells (26). The deficiency of sufficient sialic acid decoration on sperm increases their susceptibility to immune-mediated clearance within the FRT (23). Sperm desialylation is an important step in capacitation, as it enables the unmasking of glycoproteins, which in turn facilitates cell signaling transduction (27). The abnormally sialylated α1-acid glycoprotein has the capacity to induce infertility in males afflicted with a persistent inflammatory disease (28). The negatively charged sialoglycan coat of sperm renders them “invisible” and assists in their maturation and fertilization inside the FRT (29). These findings suggest that sialoglycans play a crucial role in immune modulation before fertilization.

### 3.2 Impact on the process of implantation

Endometrial receptivity and maternal immune supervision permit the process of allograft fetal extravillous cells infiltrating the maternal endometrium (30). Endometrial decidualization is an essential step in the formation of endometrial receptivity, ensuring the successful implantation of the embryo. Decidualization is the process of transforming the endometrium into decidua during the secretory phase after fertilization (31). The particular N-glycans modifications, including sialylation, of human decidual tissues, were found to have a role in endometrial decidualization (32). The terminal sialyl Lewis X (sLeX) oligosaccharide catalyzed by ST3Gal3 is markedly elevated during the secretory phase and inspires endometrial receptivity (33). Terminal sialylation occurring in the decidua and decidual secretions during early pregnancy is involved in preparing for implantation (34, 35). The glyco-code, which is located on the surface of the blastocyst, contributes a crucial role in determining compatibility with the maternal host and interspecies reproductive isolation (36). The α2,6-sialylation of E-cadherin mediated by ST6Gal1 contributes to embryo adhesion during implantation by modulating uterine lumen closure (24). The interaction between sLeX on the endometrium and the upregulated L-selectin on trophoblasts facilitates the embryo’s adhesion to the endometrial epithelium (37, 38). Adhesion mediated by L-selectin aids cytotrophoblast invasion and migration to the uterus (39). The absence of sialoglycans due to genetic ablation of CMAS in the embryo triggers the activation of complements and leads to an increased infiltration of neutrophils, ultimately resulting in embryonic mortality (13). The outcome of pregnancy is also influenced by maternal NK cells, dendritic cells (DC), macrophages, and T cells in the decidua (31). Sialoglycans play a role in the maternal immune responses that are triggered by these cells against the semiallogeneic antigens of the embryo.

### 3.3 Immune regulation during pregnancy

The trophoblastic epithelium is covered with a sialic acid-rich coating, which could potentially conceal transplanted antigens (5). In a similar manner, tumor cells exploit sialoglycans to mask themselves from immune surveillance (6), a mechanism that parallels the maternal-fetal tolerance observed during pregnancy. By antigen masking, sialoglycans impede the direct recognition of immune cells. Moreover, the indirect recognition of sialylated antigens is hindered by their interaction with Siglec-G on DC phagosomes (40). Besides their functions in immune recognition, sialoglycans also exhibit multiple immune regulatory roles (**Figure 3**). During the process of implantation, leukocytes, comprising around 65%-70% natural killer (NK) cells, 10%-20% major histocompatibility complex class II positive antigen-presenting cells (MHC II^+^ APC), a few T cells, and almost no B cells, accumulate in the uterus (41, 42). Among the APC population, around 5%-10% of hematopoietic uterine cells are CD11c^+^ DC (41, 43). During the gestation period of mice, the percentage of CD11c^+^ DC exhibits an upward trend commencing at E5.5 (embryonic day 0.5, vaginal plug observation defined as gestational day 0.5, E0.5), which corresponds to the completion of implantation. This upward trend persists until E9.5 and remains stable until E17.5 (43). This implies that semiallogeneic embryos initiate the immune responses related to DC and activate particular immunological processes to ensure their survival.

#### 3.3.1 DC-NK cell balance

DC is a potent type of APC that has the ability to activate the adaptive immune response by processing and presenting antigens to naïve T cells. Decidual DC during pregnancy promotes the differentiation of T cells into T regulatory cells (Treg) and decreases the cytotoxicity of NK cells (44). It is well understood that the interactions between NK cells and DC are crucial for maintaining control of innate and adaptive immunity. NK cells, as the most abundant type of leukocytes in the pregnant uterus, do not function as killers, but rather provide the immune regulatory roles (45). Decidual NK cells are reported to release various cytokines and chemokines to regulate cells surrounding them, contributing to placentation, vascular remodeling, trophoblast migration, and immune tolerance (46, 47). Depletion of NK cells has been documented to impede decidual development and result in early pregnancy loss (48, 49). DC expansion enhances the deleterious effects of NK cell ablation by increasing inflammation-related gene expression, immunogenic activation of DC, and imbalanced generation of anti-angiogenic signals (48). Decidual NK cells exhibit high levels of X-C motif chemokine ligand 1 (XCL1), which is recognized by its receptor XCR1 on conventional type 1 dendritic cells (cDC1), thereby recruiting cDC1 (50, 51). During early pregnancy, NK cells recruit and differentiate to decrease the immunogenicity of DCs by secreting IL-10 (**Figure 3B**) (48). Thus, balanced DC-NK cell interactions are crucial during the process of placentation. Furthermore, the dysregulation of NK cells or DC resulted in changes to the glycophenotype of the implantation sites. The placental labyrinth, which is the location where maternal-fetal exchange occurs, exhibited an alternation of α2,3-linked and α2,6-linked sialic acids after the depletion of NK cells (52). Maintaining a harmonious immune system and proper sialylation patterns are essential for successful implantation and placentation during pregnancy.

Hypersialylation provides a protective mechanism for allogeneic and xenogeneic cells by shielding them from NK immunosurveillance and NK-mediated killing (**Figure 3B**). One of the explanations is that elevated levels of sialoglycans lead to an increase in Siglecs on NK cells (53). Numerous NK cells emerge at the interface between the mother and fetus during the initial stages of pregnancy but decrease once the placenta established (54). Typically, NK cells are the predominant type of lymphocytes in decidua during normal pregnancy, while they only account for ~10% in the periphery. More than 90% of NK cells are CD56^bright^ in decidua, compared to less than 10% in the periphery (55). Siglec-7 and Siglec-9 are sialoglycan receptors found on human NK cells that suppress the immune response in a manner independent of MHC class I. Siglec-7 is highly abundant on NK cells, while Siglec-9 is absent on CD56^bright^ NK cells but is expressed on 40%–50% of CD56^dim^ NK cells. Siglec-7 is widely detected in CD56^bright^ NK cells from cord blood, but its expression varies significantly in adult peripheral blood. Approximately 10% of CD56^bright^ NK cells derived from cord blood express Siglec-9 after neuraminidase treating. Siglec-7 and Siglec-9 expression levels are elevated in CD56^dim^ NK cells from cord blood when compared to those in the adult periphery. Following neuraminidase treatment, the proportion of CD56^dim^ NK cells expressing Siglec-9 increases from 40% to 60%. CD56^dim^ Siglec-9^+^ NK cells exhibit reduced cytotoxicity but enhanced chemotactic potential. Thus, it is thought that the presence of Siglec-9 belongs to an initial occurrence during the transition from CD56^bright^ to CD56^dim^ NK cells (56). CD56^bright^ NK cells conduct immunological modulatory functions through the release of different cytokines, whereas CD56^dim^ NK cells exert cytotoxic capabilities (57). Given that a significant proportion of NK cells in the decidua are CD56^bright^ and considering the potential influence of sialoglycans on NK cell differentiation, it may be concluded that NK cells in decidua primarily perform immunological regulatory functions and depend on the presence of sialoglycans.

#### 3.3.2 Decidual T cells regulation and differentiation

Decidual T cells consist of two main subsets: CD4^+^ T cells, accounting for around 30% to 45%, and CD8^+^ T cells, comprising around 45% to 75% (58). Decidual CD8^+^ T cells express elevated amounts of Tim-3 and PD-1, which recognize PD-L1 on extravillous trophoblasts, compared to those in the periphery. This leads to antigen-specific tolerance to trophoblasts (59). Local T regulatory cells (Treg) prevent CD4^+^ T cells from lysing trophoblast cells (60). During implantation (E3.5), there is a temporary increase in CD4^+^ CD25^+^ Foxp3^+^ Treg cells in uterine draining lymph nodes. They are also found in greater levels in the uteruses of pregnant mice compared to mice in estrous (61). On NK cells and T cells, the sialoglycan receptor Siglec-9 has been found to be expressed along with inhibitory receptors like PD-1, LAG-3, and Tim-3. Their co-expression led to a reduction in the cytotoxicity of these cells (62). Additionally, sialoglycans interact with Siglec-9, which is located in proximity to the TCR-CD3 complex. This interaction inhibits TCR-mediated cell activation by reducing ZAP 70 phosphorylation by recruiting SHP-1 through the immunoreceptor tyrosine-based inhibitory motif (ITIM) phosphorylation (6, 63). As above mentioned, Siglecs like Siglec-9 and Siglec-10 within the FRT hinder the leukocytic reaction to facilitate the survival of sperm (22, 64). Glygodelin-A, which is the amniotic glycoform of placental protein 14, acts as a glycoprotein ligand for Siglecs. It promotes the transformation of T cells into Treg rather than effector T cells (**Figure 3C**) (65, 66). As interactions between Siglecs and sialoglycans act as glyco-immune checkpoints, their roles in maternal-fetal immune tolerance need further investigation. However, the dissimilarities between the Siglec family members in mice and humans restrict their *in vivo* investigations. Thus, it is necessary to develop particular techniques and methods to address this issue. In recent years, research on sialoglycans in oncology has gained considerable attention, and examining the overlapping roles of sialoglycans in both pregnancy and tumors may foster new research strategies.

## 4 The overlap relevance in tumor and pregnancy

Tumor progression is partly similar to embryo implantation. The infiltration and invasion of trophoblasts during pregnancy are governed by regulation, whereas that of tumors is characterized by an unrestricted process (67). It is unknown whether these similarities and distinctions between these two conditions contribute to the solution of their complicated issues. Understanding the mechanisms that restrict the invasion of trophoblasts has the potential for developing strategies to halt the pathological proliferation of tumor cells. Similarly, conditions like inadequate remodeling of uterine spiral arteries and recurring implantation failure may be solvable by understanding the mechanisms of tumor progression.

**Figure 4:**
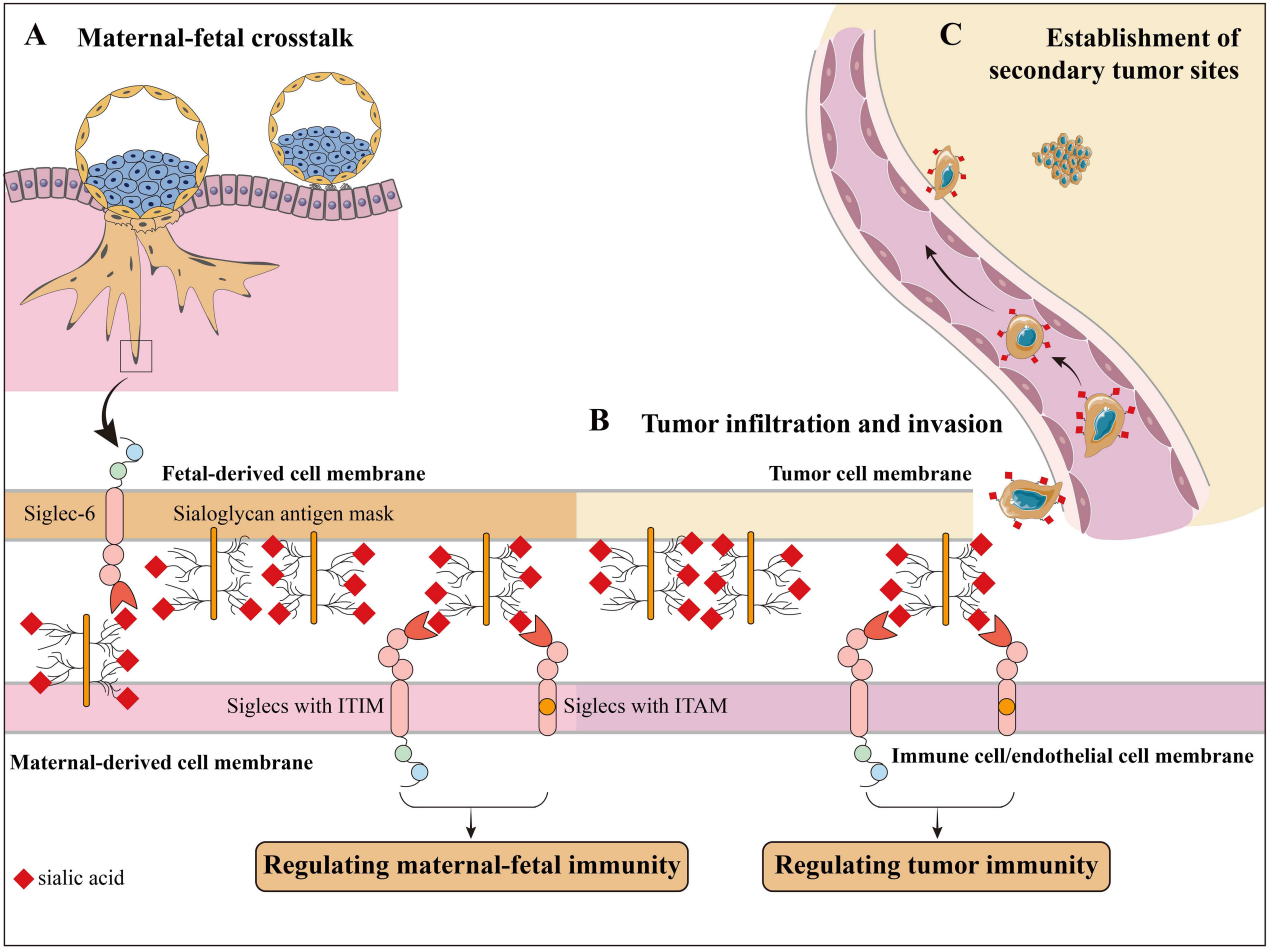
The overlapped roles of sialoglycans in pregnancy and tumor. The maternal-fetal immunity shared similar mechanisms with tumor immunity. **(A)** Sialoglycans regulate maternal-fetal immunity. **(B)** Sialoglycans are involved in tumor infiltration and invasion. For example, sialoglycans on tumor cells induce EMT and endothelial activation, allowing infiltration and invasion. **(C)** Sialoglycans help the survival of tumor cells during their bloodstream and lymphatic transportation and the establishment of secondary tumor sites.

Sialylation, a protein post-translational modification, has been researched in both pregnancy and tumors for decades. As previously discussed, sialoglycans paly crucial roles in maternal-fetal immune regulation throughout pregnancy, contributing to the survival of gametes in FRT and implantation of blastocysts (**Figure 4A**). Sialic acid is also reported to alter the conformation and flexibility of glycan chains, thus changing the protein functions (68). The presence of sialoglycans on the epidermal growth factor receptor promotes the process of epithelial to mesenchymal (ETM) transition in cancer cells (69) (**Figure 4B**). Elevated levels of sialoglycans on tumor necrosis factor receptor 1 (TNFR1) and Fas hinder apoptosis signals, allowing cancer cells to escape apoptosis as they move through the blood and lymphatic system, leading to the formation of secondary tumors (70) (**Figure 4C**). These mechanisms are also being studied in the context of pregnancy (71, 72). Therefore, it is essential to explore the overlap field of sialoglycans in tumor immunity and maternal-fetal immunity (**Figure 4**). Recent studies have demonstrated that sialoglycan inhibitors, such as anti-Siglec antibodies, are in development for cancer immunotherapy (73, 74). These inhibitors could potentially be repurposed for addressing pregnancy-related immune disorders like preeclampsia.

The presence of Siglec-6 on trophoblast cells and Siglec-5/14 on amniotic epithelium suggests that their interaction with sialylated ligands may play a role in pregnancy (75). Siglec-6, which is uniquely expressed on human placental trophoblast, regulates cell proliferation, invasion, and apoptosis during pregnancy, and its expression in normal placentas drops markedly after eight weeks gestation (76). The dynamic expression of Siglec-6 may indicate that its downregulation serves as a brake on trophoblast invasion. Interestingly, Siglec-6 is also implicated in the progression of certain cancers, such as bladder cancer, where its expression correlates with poor prognosis (77–79). This suggests that Siglec-6 could serve as a shared therapeutic target in both contexts and implies the possibility that physiologic pregnancies hold the secret to preventing cancer invasion.

Within the amniotic epithelium, there is a pair of Siglec receptors, Siglec-5 and Siglec-14. Siglec-5 serves as an inhibiting receptor. Siglec-14 functions as an activating receptor. These receptors are essential for controlling the immune response to invasive infections caused by Group B *Streptococci* (80). The Siglec-5 molecule also serves as an inhibitory immunological checkpoint in specific tumor cells, and blocking it shows potential in enhancing the T cell immune response against tumors (81). Siglec-14, which shares sequence similarity with Siglec-5, increases the lipopolysaccharide-induced production of TNFα and exhibits distinct immunological properties (82). In the human genome, *SIGLEC14* is located near *SIGLEC5* and they share a remarkably identical sequence in the coding region at the 5’-end. The fusion gene formed by *SIGLEC5* and *SIGLEC14* controls the levels of Siglec-5 and Siglec-14 (82). Due to these gene signatures, the extracellular domains of Siglec-5 and Siglec-14 are structurally similar and are capable of recognizing the same ligands. However, these two Siglecs transmit signals through the activation of ITIM and immunoreceptor tyrosine-based activation motif (ITAM), respectively. Co-culture with tamoxifen-treated breast cancer cells induces an immune response of monocytes caused by the overexpression of Siglec-14 rather than Siglec-5. One potential explanation for these changes could be that tamoxifen-induced estrogen-dependent sialoglycan alterations in breast cancer cells (83). These findings imply that tamoxifen has the ability to regulate the immune system and triggers an immunotherapy response through the interaction of sialoglycans and Siglecs. The similar functions of the paired Siglec-5/14 glyco-immune checkpoint in pregnancy and malignancies suggest that the underlying mechanisms of these two conditions are interconnected and complementary.

Likewise, it has been discovered that Siglec-10 interacts with sialoglycans on CD24 in the first-trimester placenta, indicating their involvement in maternal-fetal immune tolerance (84). Siglec-10 possesses two ITIM motifs, which transmit inhibitory intracellular signals (85). The immunosuppression mediated by the interaction of Siglec-10 and CD24, which is known as an innate immune checkpoint, is also observed in tumors. Increased expression of Siglec-10 in kidney renal clear cell carcinoma is associated with a negative outcome. Its expression is potentially orchestrated by transcription factors c-FOS and GATA1 (86). GATA1 expression reaches its highest level during the process of trophoblast attachment to the maternal endometrium. This indicates that GATA1 is involved in the attachment and implantation of the conceptus (87). The investigation of GATA1’s role in regulating Siglec-10 expression during pregnancy has not yet been conducted. Thus, understanding the regulation of Siglecs expression in tumors could offer valuable insights into their expression patterns in pregnancy.

## 5 Therapeutic potential of glyco-immune checkpoints

The immunological regulating of the maternal-fetal interface serves as a natural example of active immunotolerance, as it maintains a delicate balance between accepting the semiallogeneic fetus and defending against attacks from “non-self” antigens simultaneously (88). The discussion focused on the roles of sialoglycans in pregnancy and tumors as glyco-immune checkpoints. Although tumors during pregnancy are rare (approximately 1 in 1000 pregnancies), there have been reported cases of using immune checkpoint inhibitors (ICIs) (89, 90). ICIs, including monoclonal antibodies that target cytotoxic T-lymphocyte–associated protein 4 (CTLA4), lymphocyte activation gene 3 (LAG3), programmed cell death 1 (PD-1), and its ligand (PD-L1), are commonly used to treat multiple types of tumors (90). ICIs are designed to reestablish the immune response mediated by T cells against cancer cells. Nivolumab and pembrolizumab (anti-PD-1 drugs) are classified as pregnancy category D (positive evidence of risk) by the Food and Drug Administration (FDA), whereas ipilimumab (anti-CTLA-4 antibody) is labeled as pregnancy category C (risk cannot be ruled out). Preclinical studies and some reports show that administration of ICIs for treating tumors during peri-pregnancy period may raise the chances of pregnancy issues, premature birth, low birth weight, and even fetal death (89, 91). Nevertheless, there have been documented clinical instances showing positive outcomes related to pregnancy. Monotherapy with anti-PD-L1 or anti-CTLA4 did not lead to an increase in maternofetal adverse outcomes when compared to other anti-tumor medications. However, the concurrent administration of anti-PD-1 and anti-CTLA4 did lead to (90). Thus, monotherapy and close monitoring of both mothers and fetuses are necessary when administered. The immune-related adverse events here may result from the impaired immune tolerance caused by ICIs.

The establishment of immunological tolerance between the mother and fetus is crucial for the success of pregnancy. Immune system disorders are a typical reason for pregnancy loss. The immune checkpoints have been investigated to be potential biomarkers for recurrent pregnancy loss (92–94). As toxicities of ICIs on fertility, pregnancy, and sexuality have been revealed (95), their potentially therapeutic roles in recurrent spontaneous abortion were not investigated. Recently, agents targeting glyco-immune checkpoints have been studied (96, 97). GLIMMER-01’s phase I findings showed that E-602, a fusion protein made up of modified human sialidase and human IgG1 Fc region, effectively targeted immunosuppressive sialoglycans at the well-tolerated doses (97). Further assessment will be conducted on the monotherapy effectiveness of E-602 in individuals with non-small cell lung cancer and melanoma who are resistant to ICIs. Furthermore, receptors for sialoglycans, including Siglec-6 (77, 98), Siglec-10 (99, 100), and Siglec-15 (101–103), have been identified as targets for tumor therapy in clinical trials. Lirentelimab, an antibody targeting Siglec-8, has been investigated in autoimmune diseases such as chronic urticarial (104). No reports exist on the utilization of ICIs or glyco-immune checkpoint treatment for recurrent pregnancy loss. Further research is necessary to fully elucidate the role of sialoglycans in reproductive immunology. Large-scale clinical trials are required to investigate the safety and efficacy of targeting glyco-immune checkpoints in pregnancy-related complications, and parallel studies in cancer biology could provide valuable insights.

## 6 Perspectives and conclusions

Sialoglycans are involved in the entire pregnancy period and partially overlap in tumor immunity. Restoring balance to the sialylation process may potentially resolve pregnancy-related disorders like miscarriage and preeclampsia. Given the wide-ranging studies on ICIs, agents that target glyco-immune checkpoints might be a promising therapy for both tumor and pregnancy-related diseases. Interfering with glyco-immune checkpoints to locally regulate immune responses in FRT may serve as a novel therapy with low toxicity. However, further research is required to better comprehend the roles of sialoglycans throughout pregnancy to catch up with the level of knowledge we possess about malignancies. Targeting glyco-immune checkpoints in pregnancy must be approached with caution, as there is a delicate balance between immune tolerance and protection. Disruption of this balance could potentially result in adverse outcomes, such as increased susceptibility to infections or pregnancy complications.
